# Supplementary material for: Novel Driver Strength Index highlights important cancer genes in TCGA PanCanAtlas patients
Source: PeerJ. 2022 Aug 11;10:e13860. doi: 10.7717/peerj.13860 (PMC9375969; doi:10.7717/peerj.13860)
Supplement: Supplemental Information 2 [file peerj-10-13860-s002.zip › COHORTS/genes plots/age/2021_8_16_14_9_distribution_cohorts_males_40-44.pdf]

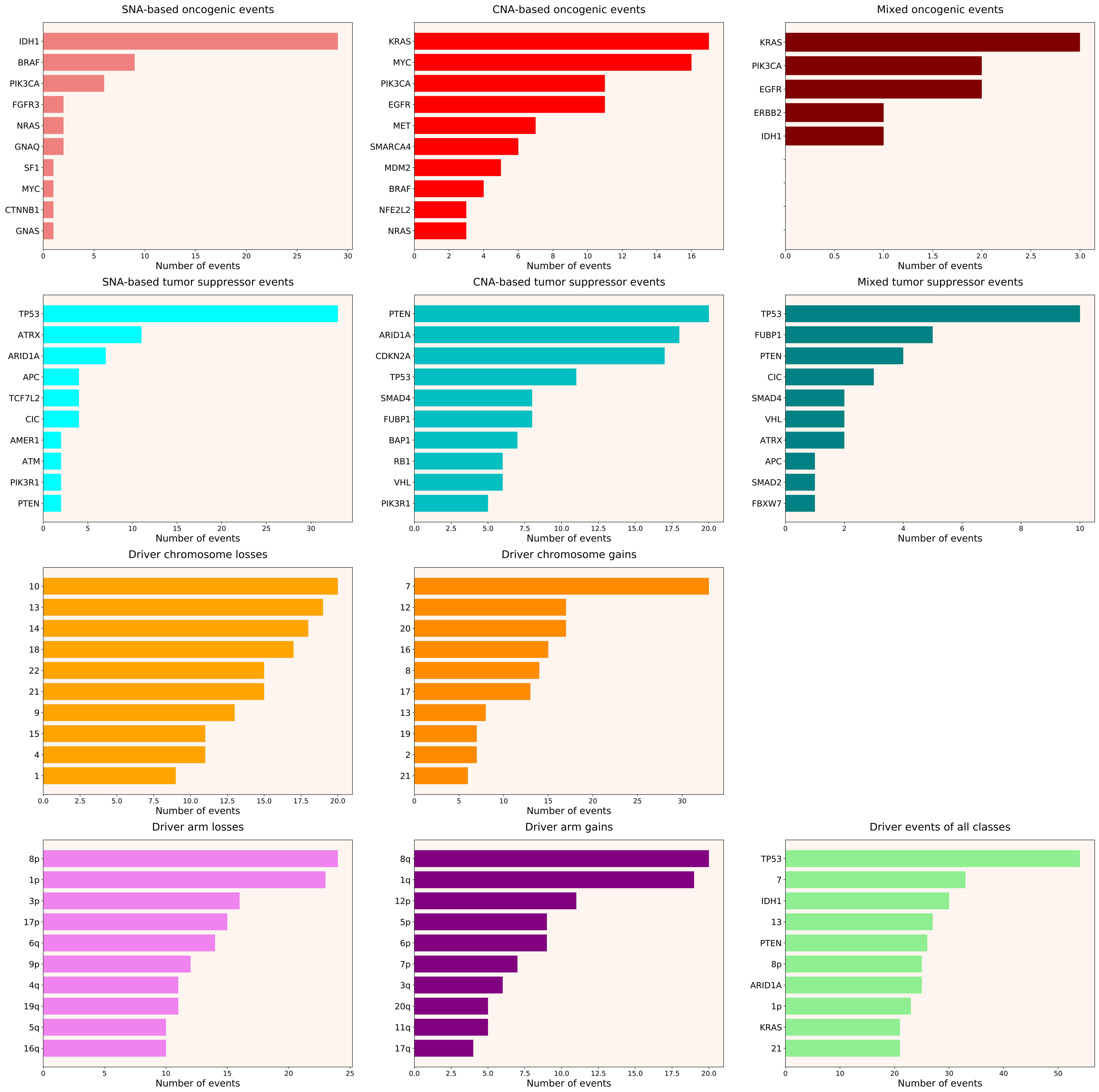
SNA-based oncogenic events
IDH1
BRAF
PIK3CA
FGFR3
NRAS
GNAQ
SF1
MYC
CTNNB1
GNAS
0 5 10 15 20 25 30
Number of events
CNA-based oncogenic events
KRAS
MYC
PIK3CA
EGFR
MET
SMARCA4
MDM2
BRAF
NFE2L2
NRAS
0 2 4 6 8 10 12 14 16
Number of events
Mixed oncogenic events
KRAS
PIK3CA
EGFR
ERBB2
IDH1
0.0 0.5 1.0 1.5 2.0 2.5 3.0
Number of events
SNA-based tumor suppressor events
TP53
ATRX
ARID1A
APC
TCF7L2
CIC
AMER1
ATM
PIK3R1
PTEN
0 5 10 15 20 25 30
Number of events
CNA-based tumor suppressor events
PTEN
ARID1A
CDKN2A
TP53
SMAD4
FUBP1
BAP1
RB1
VHL
PIK3R1
0.0 2.5 5.0 7.5 10.0 12.5 15.0 17.5 20.0
Number of events
Mixed tumor suppressor events
TP53
FUBP1
PTEN
CIC
SMAD4
VHL
ATRX
APC
SMAD2
FBXW7
0 2 4 6 8 10
Number of events
Driver chromosome losses
10
13
14
18
22
21
9
15
4
1
0.0 2.5 5.0 7.5 10.0 12.5 15.0 17.5 20.0
Number of events
Driver chromosome gains
7
12
20
16
8
17
13
19
2
21
0 5 10 15 20 25 30
Number of events
Driver arm losses
8p
1p
3p
17p
6q
9p
4q
19q
5q
16q
0 5 10 15 20 25
Number of events
Driver arm gains
8q
1q
12p
5p
6p
7p
3q
20q
11q
17q
0.0 2.5 5.0 7.5 10.0 12.5 15.0 17.5 20.0
Number of events
Driver events of all classes
TP53
7
IDH1
13
PTEN
8p
ARID1A
1p
KRAS
21
0 10 20 30 40 50
Number of events
